# Differential mRNA expression of the main apoptotic proteins in normal and malignant cells and its relation to in vitro resistance

**DOI:** 10.1186/s12935-018-0528-9

**Published:** 2018-03-05

**Authors:** Andrea Vazanova, Jana Jurecekova, Tomas Balharek, Juraj Marcinek, Jan Stasko, Anton Dzian, Lukas Plank, Pavol Zubor, Peter Racay, Jozef Hatok

**Affiliations:** 1 Department of Medical Biochemistry, Jessenius Faculty of Medicine in Martin (JFM), Comenius University in Bratislava (CU), Mala Hora 4D, 03601 Martin, Slovak Republic; 2 Clinic of Haematology and Transfusiology, JFM CU and Martin University Hospital (MUH), Kollarova 2, Martin, Slovak Republic; 3 Biomedical Center Martin, JFM CU, Mala Hora 4D, Martin, Slovak Republic; 4 Department of Pathologic Anatomy, JFM CU and MUH, Kollarova 2, Martin, Slovak Republic; 5 Clinic of Thoracic Surgery, JFM CU and MUH, Kollarova 2, Martin, Slovak Republic; 6 Department of Obstetrics and Gynecology, CU JFM and MUH, Kollarova 2, Martin, Slovak Republic

**Keywords:** Apoptosis, Resistance, Malignant cells, mRNA expression

## Abstract

**Background:**

Apoptosis plays an important role in the development and homeostasis of multicellular organisms and its deregulation may result in many serious diseases, including cancer. Now it is clear that some oncogenic mutations disrupt apoptosis, leading to tumour initiation, progression or metastasis. Here, expression of apoptotic genes in context of drug resistance was investigated.

**Methods:**

We examined total of 102 samples from leukemic patients (n = 60) and patients with solid tumours (n = 42). We used RT-PCR to determine the levels of mRNA expression and the in vitro chemoresistance of leukemic cells was evaluated using the MTT assay.

**Results:**

We found statistically significant increase in mRNA expression of all investigated proteins (p53, BAX, Bcl-2 and Bcl-XL) between the leukemia samples and leukocytes from healthy volunteers. We did not find any significant difference in mRNA levels among the solid tumour samples. Notably, we showed a significant positive correlation in both leukemic and solid tumour patient groups between *p53* and *BAX* mRNA. We found that the highest values for the *Bcl*-*2/BAX* ratio were in solid tumours in comparison to leukemic cells or normal leukocytes. Moreover, we assessed the impact of *p53* and *BAX* mRNA levels on the sensitivity of the leukemic cells to selected cytostatics.

**Conclusions:**

Elevated levels of *p53* and *BAX* mRNA may indicate cellular response to possible changes in genomic DNA integrity associated with malignant transformation. We suggest that the *BAX* gene is regulated by the p53 protein but the initiation of apoptosis through the transcription activation of *BAX* is blocked by the high levels *of Bcl*-*2*. Given that the apoptosis resistance mechanisms are different among oncological patients as well as stages of identical malignancy cases, personalized and specific combination therapy is proposed to be more effective in clinical application.

## Background

Deregulation of apoptosis (programmed cell death) is considered as one of the most important process in cancer development and progression [1] although the involvement of deregulated apoptosis in malignant transformation is not completely clear [2]. The ability of cancer cells to avoid apoptosis and continue to survive and proliferate is one of the hallmarks of cancer [3] and is a major target of cancer therapy development [4].

P53 belongs to one of the central control nodes that regulate the decisions of cells to proliferate or to undergo apoptotic program. It is well known that *p53* suppresses tumour formation and renders protection against DNA damage by inducing cell cycle arrest, DNA repair, or apoptosis [5, 6]. Technically, upon activation triggered by signals like hypoxia or radiation induced DNA damage, *p53* acts as zinc-containing transcription factor and regulates downstream genes that are involved in DNA repair, cell cycle arrest or apoptosis. Apoptosis is initiated by trans-activating proapoptotic proteins such as P53-upregulated modulator of apoptosis (PUMA), Tumor necrosis factor receptor superfamily member 6 (FAS) or Bcl-2 associated X (BAX) [7]. Moreover *p53* is capable of transcriptional repression of Bcl-2 (B-cell CLL/lymphoma 2) in various cancers including hematopoietic malignancies [8, 9]. P53-mediated regulation of the ratio of Bax versus Bcl-2 protein level can influence the fate of a cell in response to stress [10]. However *p53* is also the most frequently mutated gene in human cancer and the frequency of *p53* mutations is highly variable depending on the type of cancer [11].

Abnormalities in the bcl-2 family proteins have been considered to play an important role in some types of cancers [12, 13]. The bcl-2 associated X, apoptosis regulator gene is a member of the bcl-2 gene family and is a transcriptional target of tumor protein p53 (*p53*). BAX is a main proapoptotic protein since BAX homodimerisation and consequent pore formation in outer mitochondrial membrane is required to promote apoptotic cell death. BAX also forms heterodimers with Bcl-2 that leads to neutralisation of its apoptotic function. The absence of BAX expression in some cell lines of human haematopoietic malignancies [14] and colorectal cancers [15, 16] has been reported to result from the insertion or deletion of a single residue in the (G)8 tract within the *BAX* coding sequence. The antiapoptotic proteins which have interested in our study were B-cell CLL/lymphoma 2 and BCL2 like 1-extra large (Bcl-XL). Both antiapoptotic proteins has the BH1-3 domains arranged to expose a hydrophobic groove that is required for their pro-survival activity and binding of their proapoptotic partners [17]. Overexpression of Bcl-2 and Bcl-XL proteins is observed in many malignancies [8, 18, 19], and can result from gene amplification, increased gene transcription, chromosomal translocation and altered post-translation processing. The mentioned processes produce chemoresistant cells where the Bcl-2 antiapoptotic proteins are frequently upregulated, offsetting the function of proapoptotic proteins. Therefore, ratio between apoptotic promoters and repressors in the Bcl-2 family determines the chemosensitivity of cells to apoptotic stimuli. Finally, determining the relationship between the p53 and main apoptotic proteins (Bcl-2, Bax, Bcl-XL) in different type cells can determine the development of chemoresistance and may be estimated a targets for gene therapy in malignancy treatment.

The aim of the present study was to investigate mRNA expression levels of *Bcl*-*2, Bcl*-*XL, BAX* and *p53* mRNA in leukemic cells and correlate them with in vitro sensitivity of leukemic cells to selected cytostatics. In addition expression levels of *Bcl*-*2, Bcl*-*XL, BAX* and *p53* mRNA in solid tumour samples was investigated.

## Materials and methods

### Sample collection and processing

A total of 118 samples from three different organs and whole blood were collected from the University Hospital in Martin and Hospital in Prievidza. Approval by the local Ethics committee of the Jessenius faculty of medicine in Martin was obtained under approval number EK 1255/2013 and this study has therefore been performed in accordance with the ethical standards laid down in the 1964 Declaration of Helsinki and its later amendments. Each specimen was reviewed by two expert pathologists. After obtaining informed consent, clinical samples of whole blood (WB) were obtained from 16 healthy volunteers (median age = 30 years) and patients at diagnosis prior to treatment and during relapse prior to re-induction treatment (acute myeloid leukemia, AML = 34; acute lymphoblastic leukemia, ALL = 21; and chronic myeloid leukemia, CML = 5; median age = 42 years). Mononuclear cells (MNCs) were separated from WB by centrifugation using lymphocyte separation medium LSM 1077 (PAA Laboratories) according to the manufacturer’s protocol. Blast cells in the separated MNCs amounted to more than 80% for most patients.

Forty-two primary tumours were used in this study after surgical procedures. The solid cancers represented colorectal carcinoma (CaCo = 18), breast carcinoma (BCa = 15) and lung carcinoma (LCa = 9) with median age 58 yrs. All of the samples were obtained with the patient’s informed consent and were histologically confirmed. The size of the samples was around 75 mg and they were stored in RNAlater following the Qiagen’s protocol.

### RNA extraction and reverse transcription polymerase chain reaction (RT-PCR)

**Fig. 1:**
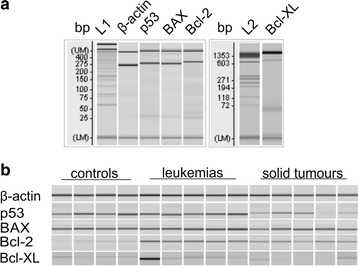
Digital gel images of representative PCR products separated by chip electrophoresis. **a** An overview of studied genes. Lane L1 represents 25 bp DNA Ladder, then follow the representative lanes for RT-PCR products for *β*-*actin, p53, BAX* and *Bcl*-*2* genes. Electrophoresis have been done using Shimadzu DNA-500 kit. Lane L2 represents ΦX174 DNA/HaeIII Marker, then follows the representative lane for *Bcl*-*XL* PCR gene product. *Bcl*-*XL* RT-PCR product was separated using Shimadzu DNA-1000 kit. **b** Representative overview of levels of mRNA expression after RT-PCR for β-actin as internal control and *p53*, *BAX*, *Bcl*-*2* and *Bcl*-*XL* in group of control samples, leukemia patients and patients with solid tumours

Total cell RNA was extracted using Trizol Reagent (Invitrogen, CA, USA) following the manufacturer’s protocol. Five micrograms of purified cellular RNA was converted to single stranded cDNA using the oligo(dT)18 primer, M-MuLV reverse transcriptase (RevertAid™ H Minus First Strand cDNA Synthesis Kit, Fermentase—EU). The cDNAs homologous with p53, Bcl-XL, BAX and β-actin (as a housekeeping gene)—positive strand RNA—were amplified for 30 cycles, using specific primers (Table 1) and an automated thermal cycler. The cycling conditions included 60 °C annealing step for 40 s, 72 °C extension step for 40 s, and 95 °C denaturation step for 20 s. For each sample, a reaction without reverse transcriptase (negative control) was performed. Positive control was obtained from the three cDNA of patient samples with positive amplification to the primer products. Both of these were simultaneously amplified by PCR and added to the strips at each assay. The PCR products (Fig. 1) of each sample were electrophoretic separated and analysed by DNA-500 or 1000 kit using a Microchip Electrophoresis System—MCE^®^-202 (Shimadzu, JAP).

**Table 1:** Products information and nucleotide sequences of primers used in amplification

Gene	Amplicon size (bp)	Primer sequence (5′–3′)
β-Actin	238	F: GGG TCA GAA GGA TTC CTA TG R: GGT CTC AAA CAT GAT CTG GG
BAX	246	F: GCC CTT TTC TAC TTT GCC AGC R: TCA GCC CAT CTT CTT CCA GAT
Bcl-2	243	F: GGC CTT CTT TGA GTT CGG TGG R: GAT AGG CAC CCA GGG TGA TGC
Bcl-XL	582	F: CTG GTG GTT GAC TTT CTC TCC R: GCT GCT GCA TTG TTC CCA TAG
p53	250	F: CCT CCT GGC CCC TGT CAT CTT R: ACC TCC GTC ATG TGC TGT GAC

*F* forward, *R* reverse

### Evaluation of sensitivity to anti-cancer drugs using the MTT assay

We used the colorimetric methyl thiazol-diphenyl-tetrazolium bromide (MTT) assay described by Mosmann [20], which is applicable to various type of living cells [21]. Briefly, MNCs of AML and CML were cultured in 96 well microculture plates for 3 days at a final concentration of 1.0 × 10^6^ cells/ml, with or without cytotoxic agents. In the present study we applied generic anticancer drugs for the following reason: long period of sample collection, most commonly used in clinical practice, different mechanisms of action and suitable for in vitro MTT assay. The 39 leukemic samples were tested with up to five drugs, depending on the quantity of cells available: cytarabine (Ara-C, 10–0.01 μg/ml), daunorubicin (Dau, 2–0.002 μg/ml), etoposide (VP-16, 50–0.048 μg/ml), idarubicin (Ida, 2–0.002 μg/ml), vincristine (Vnc, 50–0.048 μg/ml). After 72 h of incubation, 10 μl of MTT solution (5 mg/ml) was added to each microplate well and the plates were incubated for further 6 h. The produced formazan was solubilised by adding 100 μl of 10% sodium dodecyl sulphate (SDS), pH = 5.0. After an overnight incubation at 37 °C, the optical densities (OD) at 540 nm were measured using a microplate reader (Bio Rad Reader 2100). Cells incubated in culture medium alone served as a control for cell viability (untreated cells). All assays were performed in quadruplicate and mean ± SD values were used to estimate cell viability.

### Statistics

The correlation among lethal concentration (LC50) values of different drugs and groups of patients were estimated using non-parametric Wilcoxon matched-pairs signed-ranks test and Student’s T test. The statistical level of significance was set as two-tailed p ≤ 0.05. All statistical calculations were performed using the statistical package MedCalc 8.1.1 (MedCalc Inc.^®^, Belgium). Linear regression analysis was used to determine the strength of the relationship between LC50 dates and RNA expression levels using OriginPro 7 SR2 software (OriginLab Corporation, USA). The strength of this relationship was defined by using the R value (Pearson’s correlation coefficient).

## Results

### Expression of Bcl-2, Bcl-XL, BAX and p53 mRNA in leukemic samples and solid tumours

We compared the expression levels of genes coding for apoptotic proteins in different types of malignant cells. Samples were divided into three groups: controls (healthy donors), leukemias (patients with AML, ALL and CML) and tumours (solid tumours with mixed origin). We used the *ß*-*actin* gene as a control housekeeping gene. Gene expression levels were quantified using MultiNA Viewer (Shimadzu) based on the results from gel image of PCR products.

**Fig. 2:**
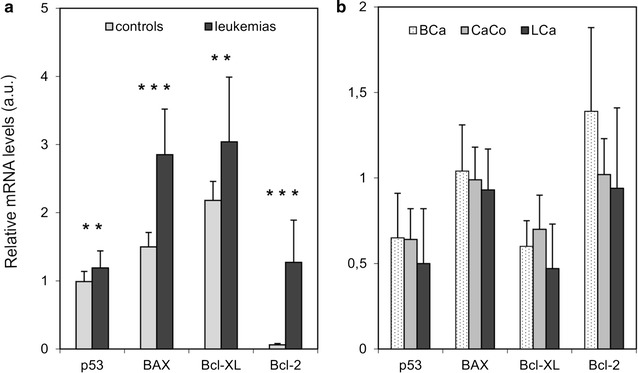
Comparison of mRNA levels of selected genes among different tumour cells. Relative mRNA levels of *p53*, *BAX*, *Bcl*-*XL* and *Bcl*-*2* gene in group of controls versus leukemia samples (**a**) and between groups of three different tumour samples—breast cancer, colorectal carcinoma and lung cancer samples (**b**). mRNA level of each gene is normalized to β-actin level. Error bars represent SD. **p < 0.01, ***p < 0.001. *CaCo* colorectal carcinoma, *BCa* breast carcinoma, *LCa* lung carcinoma

The relative expression levels of *Bcl*-*2, Bcl*-*XL, BAX* and *p53* mRNA in the leukemic samples were significantly elevated to 21.2 (p < 0.001), 1.4 (p < 0.01), 1.9 (p < 0.001) and 1.2 (p < 0.01) fold, respectively, compared to control cells. The expression levels of the examined genes from representative samples (five leukemic, five solid tumour samples) are shown in Fig. 1. Differences in the mRNA levels of leukemic samples compared to control healthy leukocytes are shown in Fig. 2a. The highest mRNA expression level in solid tumours (Fig. 2b) were obtained for the Bcl-2 protein in breast carcinoma samples. There was however a great deal of heterogeneity among these samples and statistical analysis did not show any significant differences.

### Bcl-2 to BAX ratio in leukemic samples and solid tumours

**Fig. 3:**
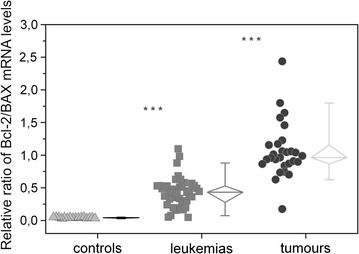
Reciprocal ratio of mRNA levels of *Bcl*-*2* and *Bax*. The lowest and the most homogenous Bcl-2/BAX ratio was detected for control samples and the highest for tumour samples. Statistical analysis have been done between controls and leukemia samples and between leukemia samples and tumours. ***p < 0.0001

It is well established that cell death or survival is connected with apoptotic protein ratio, for example *Bcl*-*2* to *BAX* ratio. For this reason we also calculated this ratio for our samples. Calculated *Bcl2/BAX* ratios are shown in Fig. 3 with notable significant difference (p < 0.0001). Statistical analysis was carried out to compare between leukemia samples and controls and between tumours and leukemias. The lowest *Bcl*-*2/BAX* ratio was detected for control samples and the highest for tumour samples.

### Correlation of p53 and BAX mRNA levels

**Fig. 4:**
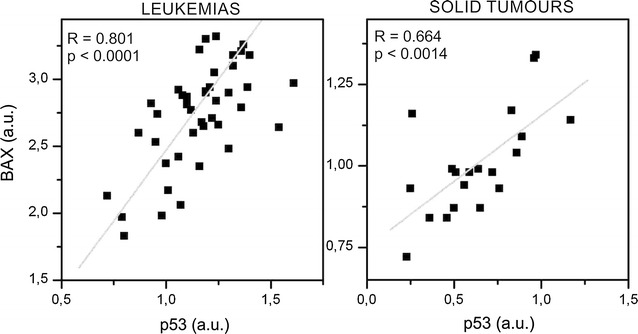
Correlation of mRNA levels of *p53* and *BAX*. Significant positive correlation in both sample groups was detected. Correlation coefficient were 0.801 for leukemia samples (n = 48) and 0.664 for solid tumours (n = 21) with p values 0.0001 and 0.0014, respectively

Based on the knowledge that the transcription of the *BAX* gene is controlled by the p53 protein we correlated transcription levels of *p53* mRNA and *BAX* mRNA in leukemia and solid tumour samples. We detected significant positive correlation in both sample groups. The p value for leukemic samples (n = 48) was p < 0.0001 and for tumour samples (n = 21) p = 0.0014 with correlation coefficients 0.801 and 0.664, respectively (Fig. 4).

### Correlation of p53, BAX, Bcl-2 and Bcl-XL mRNA levels with LC50 of cytostatics in acute leukemia samples

**Fig. 5:**
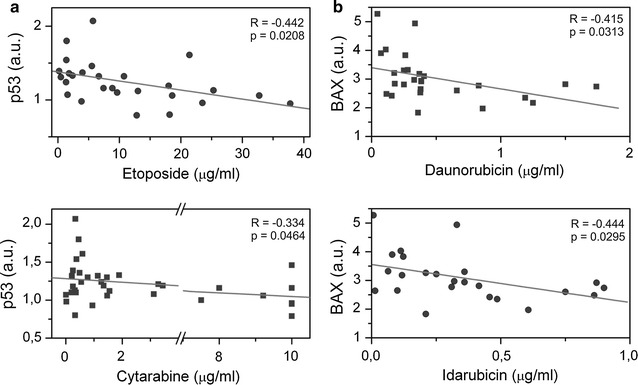
Correlation of LC50 of cytostatics with relative values of *p53* (**a**) and *BAX* (**b**) mRNA. Significant (p < 0.05) negative correlation in group of leukemia samples was detected for all four depicted cytostatics—etoposide, daunorubicin, cytarabine and idarubicin

In order to verify if there is a relationship between mRNA levels of apoptotic proteins and the sensitivity of the tested samples to cytostatic drugs, we analysed the relationship between the LC50 values of selected anti-cancer drugs and the mRNA levels of apoptotic proteins in acute leukemia samples (AML n = 34, ALL n = 5). We evaluated 39 paired experiments from AML and ALL patient samples. Statistically significant negative correlation of LC50 values of etoposide (n = 27) and cytarabine (n = 36) with the calculated *p53* mRNA levels is shown in Fig. 5a. Significant correlation for *p53* was observed for all of the tested agents except vincristine (data not shown).

Similarly we analysed the proapoptotic protein BAX. Statistically significant correlation of BAX mRNA level and two intercalating cytostatics daunorubicin and idarubicin (p = 0.031 and p = 0.029, respectively) is shown on Fig. 5b. We did not reveal any significant correlation for LC50 of cytarabine or vincristine and BAX mRNA level.

**Fig. 6:**
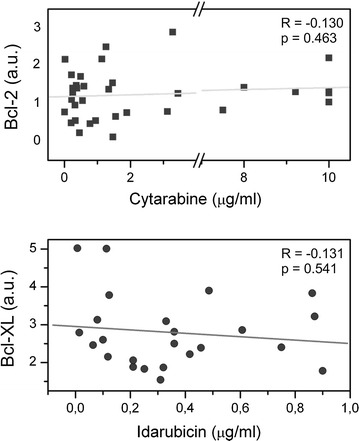
Correlation of LC50 of cytostatics with relative values of *Bcl*-*2* and *Bcl*-*XL* mRNA. No significant correlation have been observed between LC50 of cytarabine and idarubicin and levels of *Bcl*-*2* and *Bcl*-*XL* mRNA in leukemia samples

Finally, the results shown in Fig. 6, correlate sensitivity to cytarabine and idarubicine with the levels of *Bcl*-*2* and *Bcl*-*XL* mRNA, respectively. We did not see any statistical significance in the relationship between LC50 values of these cytostatics and mRNA levels of these apoptotic proteins. Though for cytarabine we could see a positive trend, thus it was the only drug which supported our hypothesis with positive correlation, although not at a significant level. Table 2 summarises the results from all comparative analyses.

**Table 2:** Statistical values of correlation between LC50 values and relative mRNA levels of apoptotic proteins in leukemic samples

Anti-cancer drug	Symbol of gene			
	p53	Bax	Bcl-XL	Bcl-2
Cytarabine	R = − 0.334 n = 36 p = *0.046*	− 0.16 36 0.350	0.011 36 0.950	0.130 34 0.463
Daunorubicin	− 0.445 26 *0.022*	− 0.415 27 *0.031*	− 0.235 26 0.246	− 0.292 23 0.176
Etoposide	− 0.442 27 *0.021*	− 0.374 28 *0.049*	− 0.203 27 0.309	− 0.269 27 0.174
Idarubicin	− 0.419 24 *0.041*	− 0.444 24 *0.029*	− 0.131 24 0.541	− 0.194 23 0.374
Vincristine	− 0.192 36 0.263	− 0.197 36 0.249	− 0.006 36 0.969	− 0.322 34 0.063

*R* correlation coefficient, *n* number of samples in the experimental group

p value is the probability level. Statistically significant p values are indicated in italic letters

## Discussion

Evasion of apoptosis represents one of the hallmark traits of malignant cells and majority of currently used antitumor drugs depend on induction of apoptosis [2]. Therefore malfunction of apoptotic mechanisms promote malignant cell survival and may attenuate the sensitivity of tumour cells to the treatment [22, 23]. Since drugs with distinct primary targets can induce apoptosis through similar mechanisms, alterations in apoptotic programs can produce inherent as well as acquired multi-drug resistance [24]. Proteins of Bcl-2 family as key players in intrinsic apoptosis have significant impact on survival and may significantly affect sensitivity of malignant cells to different types of chemotherapy [2]. Mutations rates in genes coding for apoptotic proteins such as proteins of Bcl-2 family are low [25]. Therefore majority of malignant cells have intact apoptotic machinery that if unblocked could efficiently kill them [4]. Molecular mechanism associated with evasion of mitochondrial apoptosis in malignant cells are mainly related to the over-expression of antiapoptotic proteins (based on gene translocation, gene amplification and increased stability of mRNA) or down-regulation of proapoptotic proteins (based on gene loss or gene silencing) of Bcl-2 family [16]. In accord with this, we have documented statistically significant increase of both *Bcl*-*2* and *Bcl*-*XL* mRNA as well as *Bcl*-*2/BAX* mRNA ratio in leukemic cells in comparison to the control healthy cells. Even higher value of *Bcl*-*2/BAX* ratio was documented in the cells of solid tumours. Thus we can assume that the antiapoptotic pathway is even more activated in solid tumours, which might also be strengthened by the heterogeneity (clarifying) of the cells. In our experiments, we have shown extremely elevated levels of *Bcl*-*2* mRNA in leukemic cells that was 21 times higher than the levels in control cells. One function of the antiapoptotic protein Bcl-2 is to form preferentially heterodimers with the apoptosis-inducing BAX protein. Therefore cell survival or cell death depends on the *Bcl*-*2* to *BAX* ratio [26], and high expression of Bcl-2 protein might block p53-dependent apoptosis [27]. Importance of Bcl-2 with respect to hematologic malignancies is indirectly documented by the fact that targeting of Bcl-2, by ABT-199, but not Bcl-XL is associated with promising clinical results in CLL and AML [14]. High expression of Bcl-2 correlated with poor clinical response to therapy in some of the hematologic malignancies, mainly lymphomas, CLL and AML [28–30]. Lower BAX protein expression level is frequently seen in patients with solid tumours and it is a negative prognostic factor for therapy response [31]. The study of Poeta was focused on the *BAX/Bcl*-*2* ratio as a predictive marker for acute myeloblastic leukemia [32]. A lower *BAX/Bcl*-*2* ratio (< 0.3) linked to a high-risk karyotype was identified in a group of 135 samples of different types of AML in comparison to immature lymphocytes. A higher ratio *BAX/Bcl*-*2* ratio significantly correlated with a higher complete remission rate, longer progression time and longer overall survival. In contrast Kornblau et al. observed that the level of BAX protein detected by immunoblotting was not correlated either with response to induction chemotherapy or overall survival rate in a group of 165 patients with de novo diagnosed AML. However the authors also pointed to a high *Bcl*-*2/BAX* ratio being associated with poor prognosis [29]. Guo et al. compared mRNA expression of *Bcl*-*2* and *BAX* between patients with acute leukemia and a control group (healthy donors). Expression of *Bcl*-*2* mRNA was generally higher in leukemia samples than in the control group (p < 0.05) [33]. The level of *BAX* mRNA and the *BAX/Bcl*-*2* ratio in leukemia patient samples were not significantly different to the control group values. There was also no association between the expression of the mentioned proteins in acute leukemia samples and the patient’s age and gender, percentage of blasts or FAB classification. Both the Bcl-2 protein level and the *BAX/Bcl*-2 ratio were linked to response to therapy and complete remission rate. Moreover the *BAX/Bcl*-*2* ratio significantly correlated with the overall survival rate. There was no significant relationship observed between *Bcl*-*2* and *BAX* mRNA [33].

Another goal of our experiments in studying *p53* and *BAX* transcription was their correlation in both leukemic and solid tumour samples. An increased level of *p53* mRNA indicates possible changes in genomic DNA integrity. In fact, several chromosomal abnormalities considered as major triggers for leukemic transformation have been observed in connection with acute leukemia [34, 35]. Activation of *p53* triggers the transcription of genes needed for DNA repair, cell cycle regulation and apoptosis and it is controlled at the posttranslational level [36]. Wojcik et al. compared levels of *p53, Bcl*-*2* and *BAX* mRNA between healthy blood donors and acute leukemia patients. Elevated levels of *p53* mRNA were found in 61% of ALL samples and 41% of AML samples. Also, an elevated level of *Bcl*-*2* mRNA was found in 77% of ALL patients and 41% of AML patients, while the expression of *BAX* mRNA was observed only in 46% of ALL samples and 22% of AML samples. However there was no significant correlation between *p53* and *Bcl*-*2* mRNA levels or *p53* and *BAX* mRNA levels. Interestingly, they found a significant difference between levels of *p53* mRNA in healthy blood donors (negative control) and patients with AML and ALL (p = 0.0062; p = 0.009). Similar differences were found for Bcl-2 mRNA (p = 0.003; p = 0.0092). Significant heterogeneity was observed for the *BAX* mRNA level between healthy blood donors and patients with AML and ALL (p = 0.051; p = 0.326 respectively) [37]. In our previous study we have observed positive correlation between *p53* and *BAX* mRNA in AML and ALL samples (n = 29) [8]. Our recent results showed positive correlation as well, with an even larger experimental group (n = 48). These results lead to the idea that in leukemic cells, initiation of apoptosis due to activation of *BAX* at the transcriptional level is inhibited most probably because of elevated level *Bcl*-*2* and possibly *Bcl*-*XL* transcription. This leads to the dysregulation of apoptosis and finally to the survival of malignant cells. Similarly to the leukemic samples we have observed statistically significant positive correlation between *p53* and *BAX* mRNA, indicating that the *BAX* gene is co-regulated with the p53 protein, which plays an important role in the progression of malignant diseases. We did not reveal any significant differences in mRNA expression levels among the studied proteins in the group of solid tumours but the values of high standard deviation in the results possibly reflect the heterogeneity of the tumour samples. It would be interesting to compare the obtained results with samples from corresponding healthy tissues or tissue cultures from a tissue bank. We were not successful in realising this idea. However a significant increase of *Bcl*-*2/BAX* ratio in solid tumour samples compared to leukemic samples (n = 45) indicates he importance of the Bcl-2 protein in the dysregulation of the apoptotic pathway. Similarly to the leukemia, we suppose that there is initiation of apoptosis at least at the level of activated transcription of the *BAX* gene, but at the same time there is inhibition of apoptosis because of elevated levels of Bcl-2.

Routine cytotoxic MTT assays for in vitro examination of cell resistance have a broad usage in experimental medicine. This method is mostly associated with testing the sensitivity of malignant cells to the drugs used in clinical practice [21]. It is often used with leukemic samples as well as with solid tumour samples [21, 38, 39]. In this study we evaluated correlation between LC50 values obtained from an MTT assay performed on isolated leukemic samples and the mRNA level of apoptotic proteins. Various studies have focused on this issue in different types of malignant cells to confirm the role of apoptosis in drug resistance development [40, 41]. Wu and co-workers illustrate several significant links between calculated in vitro cytotoxicity and expression levels of Bcl-2 protein, p53 induction and the induction of apoptosis [41]. Furthermore their results show that there is a cell cycle block controlled by p53 in malignant cells during chemotherapy (using adriamycine, etoposide, carboplatin, or cyclophosphamide). The strongest correlation was between chemosensitivity and induction of apoptosis, regardless of the p53 status or the expression of Bcl-2, in all of the studied cell lines. The level of Bcl-2 was associated with chemoresistance in lymphoma cell lines, but not in leukemic, ovarian and lung cancer cell lines. Our experiments have revealed positive correlation between *p53* or *BAX* mRNA and sensitivity of the leukemic cells to almost all of the tested cytostatics, except for vincristine. Subsequently, we expected a positive correlation of antiapoptotic protein mRNA level with degree of resistance. However this was observed only for cytarabine correlated with both *Bcl*-*2* and *Bcl*-*XL* mRNA levels. None of the calculated correlations was statistically significant. Although many agents activate p53 and p53 loss can attenuate drug-induced cell death, p53 is not indispensable for induction of cell death. When using a sufficient dose virtually all of the anti-cancer agents can induce apoptosis (and other types of cell death) independent of p53. The involvement of p53 in drug-induced apoptosis is determined by numerous factors including the type of agent, the dose, the type of tumour tissue and the mutational background. In short-term assays, Bcl-2 can promote resistance to a wide range of anti-cancer agents [42] and can even prevent p53-independent cell death [43]. Because Bcl-2 is considered as a general apoptosis inhibitor, these results argue for the broad importance of apoptosis in sensitivity to treatment. The associations between apoptosis and treatment sensitivity are most studied in patients with leukemia and lymphomas. In these malignancies, *p53* mutations correlate with short-term remission and with the occurrence of therapy-induced resistance [44]. Whereas in some studies direct links between mutations and insufficient response to treatment have been confirmed, in other studies, there was no association observed.

## Conclusion

The aim of this study was to determine the expression levels of genes coding for selected proteins of Bcl-2 family in leukemic as well as solid tumour samples and the expression levels of studied mRNAs in leukemic samples were compared with the levels of the same mRNAs in leukocytes from healthy donors. Furthermore we have performed correlation analysis of the expression of *BAX* and *p53* mRNA. Finally, we have performed analysis of the correlation between the levels of mRNA for selected proteins of Bcl-2 family and sensitivity of malignant cells to some anti-cancer drugs. We have shown increased levels of *p53, BAX, Bcl*-*2* and *Bcl*-*XL* mRNA in leukemic cell as well as increased *Bcl*-*2/BAX* mRNA ratio in leukemic and solid tumour cells. In addition, some significant correlation with apoptotic gene expression and sensitivity of malignant cells to chemotherapy was documented using ex vivo measurement of relative cell viability by MTT test.

We have shown that mitochondrial apoptosis is deregulated in both leukemic cells and solid tumours despite activation of *BAX* transcription via p53. We assume that apoptosis deregulation is a results of increased *Bcl*-*2/BAX* ratio observed both in leukemic and solid tumour samples. Elevated levels of mRNA of proapoptotic proteins p53 and BAX but not antiapoptotic correlated with sensitivity of leukemic cells to some chemotherapeutic drugs. Finally, leukemic and solid malignancies consists of various subtypes that respond differently to cytotoxic drugs and have a unique expression pattern of apoptosis genes therefore have a markedly different clinical outcome. The results indicate that combined information from drug sensitivity and apoptotic genes expression in a cells of individual oncological patient may provide personalized approach of the genes involved in anticancer drug resistance and become a useful tool in drug development.

## Abbreviations

ALL: acute lymphoblastic leukemia
AML: acute myeloid leukemia
Ara-C: cytarabine
BAX: Bcl-2 associated X
BCa: breast carcinoma
Bcl-2: B cell CLL/lymphoma 2
Bcl-XL: Bcl-2 like 1, extra large
CaCo: colorectal carcinoma
CML: chronic myeloid leukemia
Dau: daunorubicin
FAS: tumor necrosis factor receptor superfamily member 6
Ida: idarubicin
LC50: lethal concentration of cytostatic killing 50% of malignant cells
LCa: lung carcinoma
LSM: lymphocyte separation medium
MNCs: mononuclear cells
MTT: methyl thiazol-diphenyl-tetrazolium bromide
OD: optical densities
p53: tumor protein p53
PUMA: P53-upregulated modulator of apoptosis
RT-PCR: reverse transcription polymerase chain reaction
SD: standard deviation
SDS: sodium dodecyl sulphate
Vnc: vincristine
VP-16: etoposide
